# Multidimensional versus unidimensional pain scales for the assessment of analgesic requirement in the emergency department: a systematic review

**DOI:** 10.1007/s11739-024-03608-5

**Published:** 2024-04-25

**Authors:** Elena Crisman, Christian Appenzeller-Herzog, Senad Tabakovic, Christian Hans Nickel, Bruno Minotti

**Affiliations:** 1 Department of Internal Medicine, Kantonsspital Winterthur, Winterthur, Switzerland; 2 University Medical Library, University of Basel, Basel, Switzerland; 3 Emergency Department, Spital Wil, Wil, Switzerland; 4 Emergency Department, University Hospital Basel, University of Basel, Basel, Switzerland; 5 Emergency Department, University Hospital Basel, Basel, Switzerland

**Keywords:** Pain measurement, Multidimensional pain scales, Analgesia, Analgesic requirement, Emergency department

## Abstract

Pain is a multidimensional experience, potentially rendering unidimensional pain scales inappropriate for assessment. Prior research highlighted their inadequacy as reliable indicators of analgesic requirement. This systematic review aimed to compare multidimensional with unidimensional pain scales in assessing analgesic requirements in the emergency department (ED). Embase, Medline, CINAHL, and PubMed Central were searched to identify ED studies utilizing both unidimensional and multidimensional pain scales. Primary outcome was desire for analgesia. Secondary outcomes were amount of administered analgesia and patient satisfaction. Two independent reviewers screened, assessed quality, and extracted data of eligible studies. We assessed risk of bias with the ROBINS-I tool and provide a descriptive summary. Out of 845 publications, none met primary outcome criteria. Three studies analyzed secondary outcomes. One study compared the multidimensional Defense and Veterans Pain Rating Scale (DVPRS) to the unidimensional Numerical Rating Scale (NRS) for opioid administration. DVPRS identified more patients with moderate instead of severe pain compared to the NRS. Therefore, the DVPRS might lead to a potential reduction in opioid administration for individuals who do not require it. Two studies assessing patient satisfaction favored the short forms (SF) of the Brief Pain Inventory (BPI) and McGill Pain Questionnaire (MPQ) over the Visual Analogue Scale (VAS) and the NRS. Limited heterogenous literature suggests that in the ED, a multidimensional pain scale (DVPRS), may better discriminate moderate and severe pain compared to a unidimensional pain scale (NRS). This potentially impacts analgesia, particularly when analgesic interventions rely on pain scores. Patients might prefer multidimensional pain scales (BPI-SF, MPQ-SF) over NRS or VAS for assessing their pain experience.

**Supplementary Information:**

The online version contains supplementary material available at 10.1007/s11739-024-03608-5.

## Introduction

Pain is defined as an unpleasant sensory and emotional experience [1]. Up to 78% of patients present to the emergency department (ED) with a pain-related chief compliant [2]. Oligoanalgesia remains an ongoing issue in the acute care setting [3]. Several factors lead to inappropriate analgesia administration and consequently to under-treated pain and patient dissatisfaction [4]. The successful management of pain in the ED is fundamental to avoid long-term consequences of under-treated acute pain such as chronic pain, delayed recovery or readmission, poorer quality of life, impaired sleep, and impaired physical function [5, 6]. On the other hand, overtreatment of pain, e.g., with opioids, has become a significant global public health crisis in recent years. Opioid-related morbidity and mortality has led to significant social and economic impact [7, 8].

An accurate assessment of patients’ pain allows an appropriate treatment of pain [9]. In daily routine, numerical or descriptive unidimensional scales such as the visual analogue scale (VAS), the numeric rating scale (NRS), or the verbal rating scale (VRS) are predominantly used. Because of their quick, immediate and easy applicability, these scales have become popular tools to quantify pain intensity and pain relief [10]. These scales, however, consider only intensity as the sole characteristic. Consequently, the question arises as to how these scales can direct appropriate administration of analgesia [11–13]. Limitations of pain scales have been articulated previously. For instance, it was shown that their performance regarding prediction of desire for analgesia is poor [14, 15]. Furthermore, it was observed that pain scores might be determined rather by the emotional dimension of pain than the sensorial one [16].

It has been suggested that multidimensional pain scales address the complexity of pain sensation more adequately by including intensity, location, affective and sensory qualities [17]. They are mainly used in the context of chronic pain [18–20]. For the ED setting, there is scarce information on their use to direct analgesia [14, 21]. Because of these potential advantages compared to unidimensional pain scales, we hypothesized that multidimensional pain scales might more effectively distinguish between patients requiring analgesia and those not. This might potentially help to counteract the imbalance between oligoanalgesia and overtreatment. Therefore, the question arises if multidimensional pain scales are a more appropriate tool for assessment of analgesia in the ED. Thus, the aim of this review was to compare multidimensional with unidimensional pain scales in assessing the patient’s analgesic requirement (defined as desire for pain medication, amount of administered analgesia, or patient satisfaction) in the ED.

## Methods

The review protocol was registered on the international Prospective Register of Systematic Reviews (PROSPERO; CRD42022301926). The systematic review was conducted and reported according to the Preferred Reporting Items for Systematic Reviews and Meta-Analyses (PRISMA) statements [22].

### Eligibility criteria

Studies that included ED patients ≥ 18 years with any painful condition, of which pain assessment was performed with at least one unidimensional pain scale (VAS, NRS, VRS, or faces pain scale/FPS), and at least one multidimensional pain scale were eligible. Studies analyzing the desire of patients for analgesia, the amount of administered analgesia, or patient satisfaction according to the pain management scales were included. Non-original articles (i.e., editorials, letters and reviews), meta-analyses, case-reports, conference abstracts, and trial registry entries were excluded.

### Search strategy

Embase (embase.com), MEDLINE (Ovid), the cumulative index to nursing and allied health literature (CINAHL, EBSCOhost) and PubMed Central (US National Library of Medicine) were searched from inception to 22th November 2021. The search strings were composed by an information specialist (C.A.-H.) and peer-reviewed by a second information specialist (Hannah Ewald; see *SearchStrategies* as supplemental material). The bibliographic database search used database-specific subject headings and text word synonyms for ED, multidimensional, and unidimensional pain scales for studies on adult patients. In CINAHL and PubMed Central, names of pain scales were searched in full text. No limits, language or publication date restrictions were applied. To complement the database searches, both the cited and the citing literature of included articles were extracted from lens.org, Scopus, and the Web of Science and screened for eligibility.

### Outcome measures

Desire for analgesia was defined as the primary outcome. Amount of administered analgesia, and/or patient satisfaction were the secondary outcomes. A narrative synthesis of the data was adopted to meet the review aims. The narrative synthesis of the findings from the included studies considered the type of intervention (pain scales used), target population characteristics, and type of outcome. Additional outcomes of interest were reported during the data extraction process.

### Data collection process

Duplicate records were removed before screening using EndNote 20 and the Bramer method [23]. Two authors (E.C. and B.M.) independently screened the titles and abstracts of all search results. Full texts of selected records were retrieved and independently reviewed for eligibility by the same authors. The same screening method was applied to records identified from backward and forward citation tracking. Discrepancies were resolved by discussion between the reviewing authors. The following data were extracted from included full texts and entered into a standardized Microsoft Excel (Microsoft Corporation, 2016) form: authors and year of publication, country of study, study design, study population, triage category, chief complaint at admission, unidimensional scale(s) with score, multidimensional scale(s) with score, desire of analgesia/pain medication, amount of administered analgesia, and patient satisfaction. The same authors independently extracted data and resolved discrepancies by discussion.

### Quality assessment

We used the Cochrane ROBINS-I tool (Risk Of Bias In Non-randomized Studies of Interventions) to assess risk of bias of observational studies [24]. ROBINS-I assessed baseline and time-varying confounding, co-interventions, selection bias, classification bias, missing data, and bias in outcome measurement. Two authors (E.C. and B.M.) independently assessed the risk of bias of the included studies. Discrepancies were resolved by consensus. Results were reported through graphical representation of bias for each outcome [25].

## Results

### Study selection

**Fig. 1:**
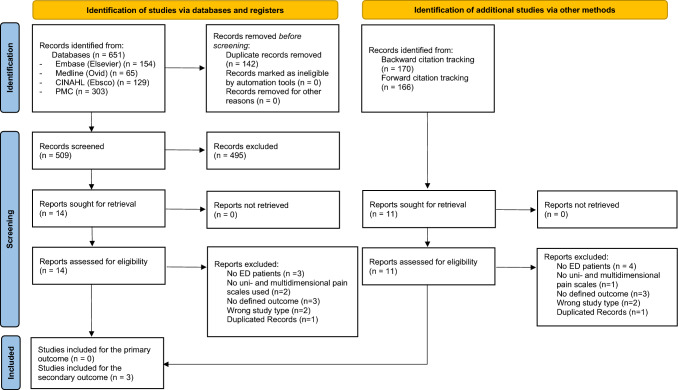
PRISMA flow chart

The database search returned 651 records of which 142 duplicates were removed. 509 records were analyzed through title and abstract screening and 495 records were excluded, with a total of 14 records remaining for full text screening [26–38]. Additional 336 papers were retrieved by citation tracking, of which 11 full-text records were assessed [9, 29, 39–47]. None of the studies met the criteria for the primary outcome. Three studies were included for analysis of the secondary outcomes [28, 30, 34] (Fig. 1).

### Study characteristics

Given the heterogeneity of study methods and outcome measures, statistical comparisons and syntheses between included studies were not possible. None of the studies analyzed desire for analgesia (primary outcome). One study analyzed the amount of administered analgesia in the context of opioid administration and prescription [30]. Two studies referred to patient satisfaction expressed as pain scale preference [28, 34]. The main characteristics of the included studies are summarized in Table 1. All studies were monocentric and prospective. VAS [34] or NRS [28, 34] were used as unidimensional pain scale, whereas the short form (SF) of the Brief Pain Inventory (BPI) [28, 34] or the McGill Pain Questionnaire (MPQ) [34], and the Defense and Veterans Pain Rating Scale (DVPRS) [30] were used as multidimensional pain scale. None of the studies compared the same pain scales (VAS vs BPI-SF and MPQ-SF [34], NRS vs BPI-SF [28], NRS vs DVPRS [30]). All outcomes are reported in Table 2.

**Table 1:** Characteristics of the included studies

Authors	Martinez et al. [34]	Im et al. [28]	Sheikh et al. [30]
Publication year	2011	2020	2021
Country of study	Brazil	USA (MA)	USA (FL)
Study design	Monocentric, prospective	Monocentric, prospective, cross-sectional	Monocentric, prospective
Study population	*n* = 20	*n* = 100	*n* = 389
	15 males [75%] median age 28 [range 19–64]	30 males [30%]	199 males [51%]
	5 females [25%]	70 females [70%]	190 females [49%]
	Median age 38 [range 27–80]	Mmean age 38 [range 18–81]	Median age 52 [IQR 38–60]
Triage category	n.a	n.a	Emergency Severity Index:
			1—Immediate: 2 (1%)
			2—Emergent: 201 (52%)
			3—Urgent: 173 (45%)
			4—Semiurgent: 9 (2%)
			5—Nonurgent: 0 (0%)
Chief complaint	Musculoskeletal pain	Chest pain: 21.4%	n.a
		Abdominal pain: 52%	
		Musculoskeletal pain: 21.4%	
		Multiple pain: 5.1%	
		Acute pain: 69%	
Unidimensional scale used with score	VAS: no values	NRS (median): 7 (IQR 5–8)	NRS (median): 8 (IQR 4–10)
Multidimensional scale(s) used with score(s)	BPI-SF, MPQ (no values)	BPI-SF Median values:	DVPRS Median score: 8 (IQR 5–9)
		Pain Severity Index (IQR) 21 (15–28) on a 0–40 scale	
		Pain Interference Index (IQR) 36 (24–50) on a 0–70 scale	
		Total BPI-SF score (IQR) 57 (43–73) on a 0–100 scale	

*USA* United States of America, *VAS* visual analogue scale, *NRS* numeric rating scale, *BPI-SF* brief pain inventory—short form, *MPQ* McGill pain questionnaire, *DVPR* defense and veterans pain rating scale, *IQR* interquartile range, *MA* Massachusetts, *FL* Florida

**Table 2:** Findings of the included studies (primary and secondary outcomes)

Studies	Martinez et al. [34]	Im et al. [28]	Sheikh et al. [30]
Desire of analgesia	n.a	n.a	n.a
Amount of administered analgesia	n.a	n.a	148 (38%) ED opioid therapy
			47 (12%) opioid prescription
			Increase NRS severity predicted ED opioid administration (OR 2.7)
Patient satisfaction	Scale preference:	Scale Preference:	n.a
	BPI-SF 50%	73% BPI-SF better than NSR	
	MPQ-SF 10%	14% no difference	
	VAS 40%	13% BPI-SF worse than NSR	
		Association between black race and BPI-SF preference	
		Association between musculoskeletal pain and BPI-SF preference	
Additional outcomes of interest	Duration of assessment (mean): BPI-SF 3 min, MPQ 4 min, VAS 1 min	Duration of assessment (mean): BPI-SF 3 min 47 s (SD 1 min 35 s)	Distribution of pain scores:
	0% VAS preference in the rheumatologic outpatient clinic for patients and caregivers, so as by caregivers in the orthopedic ward		NRS: low 39%, moderate 21%, high 40%
	100% BPI preference in the orthopedic ward for caregivers		DVPRS: low 39%, moderate 29%, high 32%
			45% of patients admitted after revisit

Note: Pain Level low = 0–3, moderate = 4–7, high = 8–10

*ED* emergency department, *OR* odds ratio, *BPI-SF* brief pain inventory—short form, *MPQ-SF* McGill pain questionnaire—short form, *VAS* visual analogue scale, *NRS* numeric rating scale, *SD* standard deviation, *DVPRS* defense and veterans pain rating scale

### Amount of analgesia

One study analyzed the use of NRS and DVPRS, comparing sociodemographic and treatment data in patients revisiting the ED within 30 days of an index visit [30]. Of the 389 patients enrolled, 259 (67%) of patients returned because of pain, 129 (51%) for acute pain, and 48 (18%) for chronic pain. In the group of patients returning to the ED for pain, 119 (46%) reported use of opioid medications at home. The results of the two scales were positively correlated, but the DVPRS was better than the NRS at differentiating between moderate and severe of pain, leading to an 8% reduction in the scores for severe pain. For both scales, there was a positive correlation observed between pain scores and the proportion of patients who received opioid treatment in the ED, as well as those who were prescribed opioids upon discharge. However, the authors concluded that the use of the DPVRS could potentially reduce opioid administrations and prescriptions, because of the better discrimination between moderate and severe pain.

### Patient satisfaction

Two studies showed a preference of patients for the BPI-SF (50–73%) in comparison with the VAS, NRS, or the MPQ-SF [28, 34]. Furthermore, associations between race and BPI-SF preference (black patients 96% vs white patients 69%) and between type of pain and BPI-SF preference were shown, with musculoskeletal pain being associated with the highest preference for the BPI-SF. We surmise that the study of Martinez et al. [34] is lacking information on the use of the SF of the scales used (MPQ and BPI), because due to the reported time required to administer the pain scale, only the use of the SF would have been possible.

### Additional outcomes of interest

The two studies analyzing patients’ preference both used the BPI-SF [28, 34]. Time required to administer the pain scale was similar (3 min vs 3 min and 47 s ± 1 min and 35 s). In the study by Martinez et al. [34] that considered also other settings than the ED, none of the patients in the rheumatologic clinic as well as none of the caregivers in the orthopedic ward preferred the VAS. In the orthopedic ward, all caregivers preferred the BPI-SF.

### Quality assessment

**Fig. 2:**
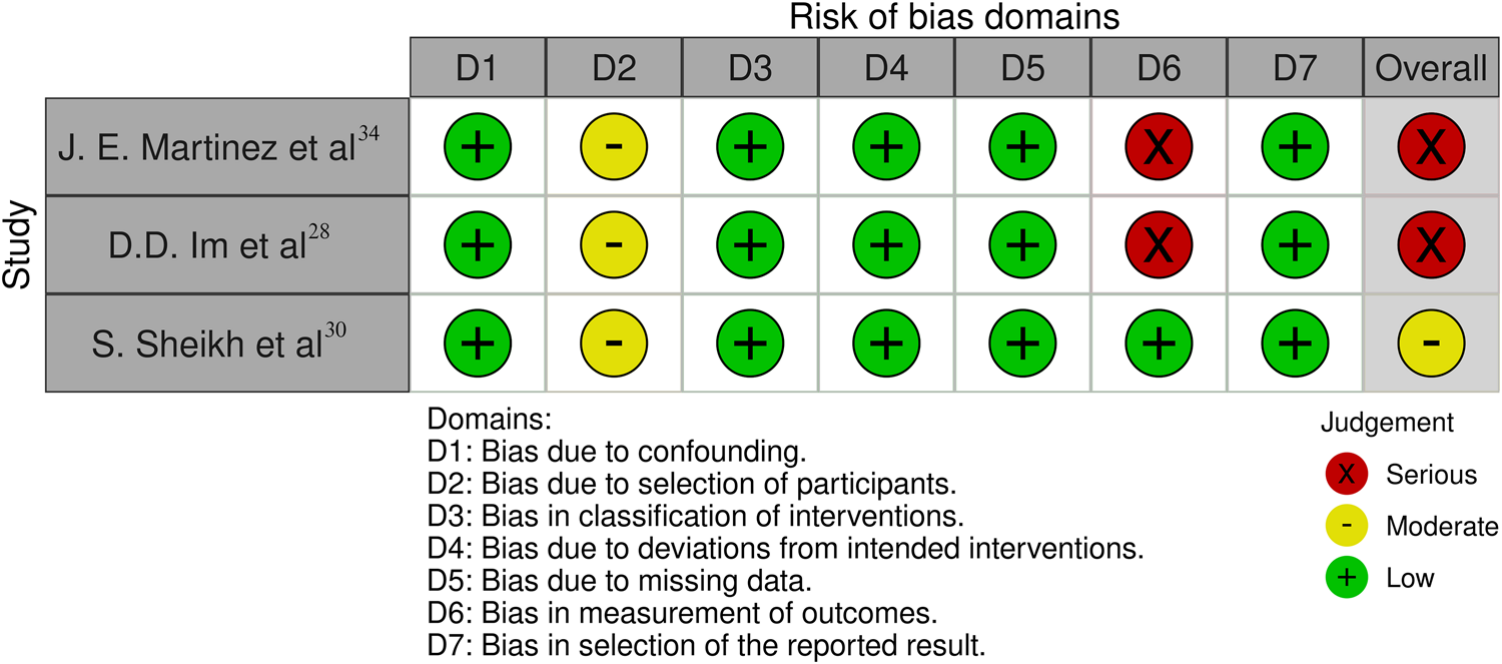
Risk of Bias analysis (Cochrane ROBINS-I tool)

The two studies analyzing patients’ preference [28, 34] presented serious risk of bias, because of lacking information concerning outcome assessment, i.e., lacking a description of how preference was determined. The study of Sheikh et al. showed moderate risk of bias including only patients revisiting the ED [30]. Risk of Bias analysis is summarized in Fig. 2.

## Discussion

This systematic review compared multidimensional with unidimensional pain scales to assess patients’ analgesic requirement in the ED. No study was found for the primary outcome, i.e., desire for analgesia. Three studies met the inclusion criteria for secondary outcomes. In one of the three included studies, a multidimensional pain scale (DVPRS) identified more patients with moderate instead of severe pain compared to a unidimensional pain scale (NRS). The authors speculated that, if analgesia was based on the score of the pain scale (as commonly specified in pain management protocols), use of DVPRS could potentially reduce opioid treatment and prescriptions [30]. In the other two studies, patients preferred multidimensional (BPI-SF, MPQ-SF) compared to unidimensional (NRS) pain scales to assess their pain experience [28, 34].

Even though the development and use of multidimensional tools to improve pain control in the acute setting was previously mentioned [11], the corresponding literature was found to be scarce. This highlights the limited use of multidimensional pain scales in the ED as well as the poor consideration of a pragmatic outcome such as patient’s analgesic requirement. Despite considering other outcomes, no other studies compared the use of multidimensional with unidimensional pain scales in the ED.

The question arises as to whether a pain scale should be used at all. For example, in daily practice, ED clinicians will frequently prescribe analgesia based on reaching a certain score on a pain scale or their own judgment of adequacy, rather than until the patient states to feel comfortable [48, 49]. This approach may stem from a reluctance to acknowledge patient-reported scores in pain assessment, as ED staff often believe they can determine the correct pain score [13]. However, even if a correct pain score exists, staff and patients differ in their estimates [50]. Nevertheless, despite the absence of an ideal pain tool for ED patients, the lack of pain assessment has been identified as one of the most problematic barriers to achieve optimal pain control [51].

The rare use of multidimensional pain scales in the ED might be explained by the length of questionnaires. However, short forms have been developed that take an average of 3–4 min to be performed. It is unknown whether this is reasonable for an ED population. The BPI-SF, for example, has been tested in one ED for certain facets of feasibility [28]. Two parameters were investigated: the time needed to complete the questionnaire, and the patient’s preference for BPI-SF versus NRS. The authors found that the mean time to complete the form was less than 4 min and that 73% of patients preferred BPI-SF over NRS. In addition, the severity of pain as assessed on the two scales was found to correspond with each other. As a result, the authors concluded that BPI-SF could be used instead of NRS to provide a more comprehensive measurement of pain. However, neither the usefulness nor the impact of BPI-SF on pain management were investigated. The BPI-SF has been used otherwise in various acute pain conditions, such as acute low back pain [52] and post-operative pain [53]. It considers two dimensions of pain: severity and its impact on daily functions. Thus, by measuring the interference of pain with the patient’s ability to perform daily tasks, BPI-SF provides a more objective measure than subjective pain ratings alone. Therefore, the use of BPI-SF could potentially lead to an improvement in the evaluation of pain. However, pain interference is typically assessed weeks after the onset of symptoms [52, 53], so that its significance during the initial hours remains unknown. In addition, it appears that pain severity initially correlates with pain interference [28], raising questions about the added value of assessing interference. An intriguing alternative perspective would be to target analgesia based on interference rather than intensity. For instance, in acute musculoskeletal pain in which restoring functionality might be the primary goal, scales evaluating daily function may be more suitable than those that focus solely on pain relief. In fact, patients with musculoskeletal pain in this review had the highest proportion preferring the BPI-SF as compared to patients with chest pain or abdominal pain [28]. The question arises as to whether a specific pain scale should be used for a particular pain condition. Numerous multidimensional pain scales have been developed within this purpose. For example, organ-related pain scales such as the Ocular Pain Assessment Survey (OPAS) [54], the Neck Pain and Disability Scale [55], or the ROwan Foot Pain Assessment Questionnaire (ROFPAQ) [56]. Other examples are disease-related pain scales, such as the Sickle Cell Disease Pain Burden Interview-Youth (SCPBI-Y) [57], or the Rheumatoid Arthritis Pain Scale (RAPS) [58]. However, all these scales were developed for longer-term outcomes, and not for the ED setting. For instance, in one study, the subscale examining pain interference (in vocational, social, and family functioning) of the multidimensional pain inventory (MPI), a multidimensional pain scale, identified patients developing persistent oro-facial pain who experienced unfavorable outcomes (pain-related disability measured with the Graded Chronic Pain Scale) [59]. The authors concluded that MPI might have a potential clinical relevance by helping to identify such individuals. However, even if such scales could aid in managing the follow-up of ED patients, they would not assist in immediately identifying patients in need of analgesia. It is of paramount importance to detect pain in ED patients and, accordingly, to provide an adequate amount of analgesia. Therefore, the ideal pain scale should not only identify patients in pain, but also those who require analgesia. Future research might prioritize the development of a new, possibly multidimensional pain scale for the ED setting by defining the “need for analgesia” as a measurable outcome. In addition, “adequacy of analgesia” should be defined as well, such as when a patient experiences pain relief, e.g., feels comfortable.

## Limitations

The comparison among the three studies is limited by their heterogeneity in endpoints, pain scales and enrolled patient cohorts. In addition, none of the studies compared unidimensional with multidimensional pain scale in a randomized design. This might have answered the hypothesis that multidimensional pain scales could more effectively distinguish between patients requiring analgesia and those not. The study of Sheikh et al. considering administration and prescription of analgesia with NRS and DVPRS used both scales alongside. Accordingly, it was not possible to directly compare administration and prescription of analgesia based on the pain scale used. In addition, they only included patients revisiting the ED leading possibly to selection bias. The two studies assessing patients’ preference had a high risk of bias. Finally, they did not specify which kind of pain experienced these patients. In addition, we assumed that patients’ preference for a pain scale would match patient satisfaction, which might not be the same. The study of Martinez et al. was limited by its small and male-biased sample size, which may have compromised its validity. The study of Im et al. was biased towards female participants. Finally, we have emphasized that pain assessment is the cornerstone of pain management. However, it is important to acknowledge that there might be other aspects to consider, such as the pure description of the pain experience itself, independent of pain management.

## Conclusions

In the ED setting, a multidimensional pain scale (DVPRS) might be able to discriminate between patients with moderate and severe pain more effectively when compared to a unidimensional pain scale (NRS). This suggests that the use of multidimensional pain scales may have implications in pain management, especially for patients potentially misclassified with severe pain by unidimensional pain scales. Based on the little heterogenous available data, patients have shown a preference for multidimensional pain scales such as BPI-SF, and MPQ-SF compared to NRS or VAS for assessing their pain experience.

### Supplementary Information

Below is the link to the electronic supplementary material.

Supplementary file1 (PDF 458 KB)
