# Supplementary material for: Multidimensional versus unidimensional pain scales for the assessment of analgesic requirement in the emergency department: a systematic review
Source: Intern Emerg Med. 2024 Apr 25;19(5):1463–71. doi: 10.1007/s11739-024-03608-5 (PMC11364591; doi:10.1007/s11739-024-03608-5)
Supplement: Supplementary file 1 — Supplementary file1 (PDF 458 KB) [file 11739_2024_3608_MOESM1_ESM.pdf]

Search strategies

Embase.com

(20211122; 154 hits)

('Emergency medicine'/de OR 'emergency health service'/de OR 'hospital emergency service'/de OR 'emergency ward'/de OR 'emergency nursing'/de OR 'emergency treatment'/de OR 'emergency care'/de OR (((emergency OR emergencies OR trauma OR accident OR urgency OR urgencies) NEAR/3 (medicine OR service* OR ward* OR unit* OR room* OR department* OR center* OR centre* OR dispensary OR nursing OR treatment* OR therapy OR therapies OR care)) OR emergicenter* OR emergicentre* OR ED OR (acute NEXT/2 care)):ab,ti)

AND

('behavioral pain scale'/de OR 'brief pain inventory'/de OR 'carpal tunnel questionnaire'/de OR 'constant murley (score)'/de OR 'fear-avoidance beliefs questionnaire'/de OR 'mcgill pain questionnaire'/de OR 'memorial pain assessment card'/de OR 'midas (migraine)'/de OR 'neck disability index'/de OR 'patient rated wrist evaluation'/de OR 'shoulder pain and disability index'/de OR 'west haven yale multidimensional pain inventory'/de OR 'multidimensional pain inventory'/de OR 'pain stages of change questionnaire'/de OR 'screener and opioid assessment for patients with pain'/de OR 'screener and opioid assessment for patients with pain revised'/de OR 'graded chronic pain scale'/de OR 'chronic pain grade questionnaire'/de OR 'chronic pain grade scale'/de OR 'dallas pain questionnaire'/de OR 'defense and veterans pain rating scale'/de OR 'neck pain and disability scale'/de OR 'core outcome measures index'/de OR 'neck bournemouth questionnaire'/de OR 'leeds assessment of neuropathic symptoms and signs pain scale'/de OR 'leeds assessment of neuropathic symptoms and signs scale'/de OR 'leeds assessment of neuropathic symptoms and signs'/de OR 'douleur neuropathique 4 questionnaire'/de OR 'douleur neuropathique 4 questions'/de OR 'douleur neuropathique 4'/de OR 'pain assessment in advanced dementia scale'/de OR 'pain assessment in advanced dementia'/de OR 'abbey pain scale'/de OR 'critical care pain observation tool'/de OR (multidimensional OR multi-dimensional OR 'behavioral pain scale' OR 'behavioural pain scale' OR 'behavioral pain rating scale' OR 'behavioural pain rating scale' OR 'behavior pain scale' OR 'behaviour pain scale' OR 'behavior pain rating scale' OR 'behaviour pain rating scale' OR BPS OR BPRS OR 'brief pain inventory' OR BPI OR 'carpal tunnel questionnaire' OR 'carpal tunnel syndrome questionnaire' OR CTQ OR 'CTS questionnaire' OR (constant NEXT/2 (murley OR scale OR score)) OR CMS OR 'fear-avoidance beliefs questionnaire' OR 'fear-avoidance belief questionnaire' OR 'fear-avoidance questionnaire' OR 'FAB questionnaire' OR FABQ OR 'mcgill pain' OR 'mcgill score' OR 'mcgill test' OR MPQ OR SFMPQ OR 'memorial pain assessment' OR 'memorial pain card' OR 'Migraine Disability Assessment' OR midas OR 'neck disability index' OR 'neck pain disability index' OR 'patient rated wrist evaluation' OR 'PRWE score' OR 'shoulder pain and disability index' OR SPADI OR WHYMPI OR MPI OR MSS OR PEG OR MCPAC OR MAPS OR 'Pain Locus of Control Scale' OR MPRCQ OR MPRCQ2 OR 'pain stages of change questionnaire' OR PSOCQ OR 'American Pain Society Pain Taxonomy' OR AAPT OR 'screener and opioid assessment for patients with pain' OR SOAPP OR MOPAT OR 'Overall benefit of analgesic score' OR OBAS OR MMPAP OR 'Integrated Pain Quantification Index' OR IPQI OR 'UAB Pain Behavior Scale' OR 'Pain and discomfort module' OR PDM OR 'Pain Experience Scale' OR 'graded chronic pain scale' OR GCPS OR 'chronic pain grade questionnaire' OR CPGQ OR 'chronic pain grade scale' OR CPGS OR 'Breakthrough Pain Assessment Tool' OR BAT OR 'Richards Assessment of Pain' OR RAP OR 'Mainz Pain Staging System' OR MPSS OR 'Composite Pain Index' OR CPI OR 'dallas pain questionnaire' OR 'Clinically Aligned Pain Assessment' OR CAPA OR 'Comprehensive Pain Evaluation Questionnaire' OR CPEQ OR 'Survey of Pain Attitudes' OR SOPA OR 'Multiaxial Assessment of Pain' OR 'Multi-Facet Pain Survey' OR MFPS OR 'defense and

veterans pain rating scale' OR DVPRS OR 'Pain Monitor' OR 'Short Form-36 Bodily Pain Scale' OR 'SF-36 Bodily Pain Scale' OR 'SF-36 BPS' OR 'Pain Impact Questionnaire' OR PIQ OR 'Victorian Institute of Sports Assessment Achilles Questionnaire' OR VISA-A OR 'ROwan Foot Pain Assessment Questionnaire' OR ROFPAQ OR J-MAP OR 'Sickle Cell Disease Pain Burden Interview-Youth' OR SCPBI-Y OR MHPQ OR 'Ocular Pain Assessment Survey' OR OPAS OR 'Vulvar Pain Assessment Questionnaire' OR 'Migraine Tension-Type Headache and Neck Pain' OR M-TTH-NP OR 'neck pain and disability scale' OR NPDS OR 'core outcome measures index' OR COMI OR 'neck bournemouth questionnaire' OR NBQ OR BQ OR 'Neuropathy Pain Scale' OR PainDETECT OR 'leeds assessment of neuropathic symptoms and signs' OR LANSS OR 'douleur neuropathique 4' OR DN4 OR 'Post abdominal surgery pain assessment scale' OR PASPAS OR 'Edmonton Classification System for Cancer Pain' OR 'pain assessment in advanced dementia' OR PAINAD OR 'abbey pain scale' OR APS OR 'Checklist of Nonverbal Pain Indicators' OR CNPI OR 'Geriatric Pain Measure' OR GPM OR GMPI OR 'Rheumatoid Arthritis Pain Scale' OR RAPS OR 'Measure of Intermittent and Constant Osteoarthritis Pain' OR ICOAP OR MPOPS OR 'critical care pain observation tool' OR CPOT OR 'Angle Labor Pain Questionnaire' OR A-LPQ):ab,ti)

AND

('faces pain scale'/de OR 'visual analog scale'/de OR 'numeric rating scale'/de OR 'verbal rating scale'/de OR 'verbal pain score'/de OR 'verbal descriptor scale'/de OR (unidimensional OR uni-dimensional OR monodimensional OR mono-dimensional OR 'faces pain scale' OR FPS OR FACES OR Wong-Baker OR 'visual analog scal*' OR 'visual analogue scal*' OR VAS OR 'numeric rating scale' OR 'numeric pain rating scale' OR 'numerical pain rating scale' OR 'numerical rating scale' OR NRS OR NPRS OR vNRS OR 'analog pain scale' OR 'analogue pain scale' OR 'verbal rating scale' OR VRS OR 'verbal pain score' OR VPS OR 'verbal descriptor scale' OR VDS OR 'verbal response scale'):ab,ti)

NOT (juvenile/exp NOT adult/exp) NOT (('animal'/de OR 'animal experiment'/exp OR 'nonhuman'/de) NOT ('human'/exp OR 'human experiment'/de))

Search narrative

- Instead of a general "pain assessment/measurement" concept, we chose to use two concept for specific pain assessment scale names: One for multidimensional scales and one for unidimensional scales
- Many of the scales are indexed by Emtree candidate terms (instead of regular Emtree terms)

Ovid Medline

(20211122; 65 hits)

(Emergency medicine/ OR emergency medical services/ OR exp emergency service, hospital/ OR emergency nursing/ OR emergency treatment/ OR (((emergency OR emergencies OR trauma OR accident OR urgency OR urgencies) ADJ3 (medicine OR service* OR ward* OR unit* OR room* OR department* OR center* OR centre* OR dispensary OR nursing OR treatment* OR therapy OR therapies OR care)) OR emergicenter* OR emergicentre* OR ED OR (acute ADJ2 care)).ab,ti.)

AND

((multidimensional OR multi-dimensional OR behavioral pain scale OR behavioural pain scale OR behavioral pain rating scale OR behavioural pain rating scale OR behavior pain scale OR behaviour pain scale OR behavior pain rating scale OR behaviour pain rating scale OR BPS OR BPRS OR brief pain

inventory OR BPI OR carpal tunnel questionnaire OR carpal tunnel syndrome questionnaire OR CTQ OR CTS questionnaire OR (constant ADJ2 (murley OR scale OR score)) OR CMS OR fear-avoidance beliefs questionnaire OR fear-avoidance belief questionnaire OR fear-avoidance questionnaire OR FAB questionnaire OR FABQ OR mcgill pain OR mcgill score OR mcgill test OR MPQ OR SFMPQ OR memorial pain assessment OR memorial pain card OR Migraine Disability Assessment OR midas OR neck disability index OR neck pain disability index OR patient rated wrist evaluation OR PRWE score OR "shoulder pain and disability index" OR SPADI OR WHYMPI OR MPI OR MSS OR PEG OR MCPAC OR MAPS OR Pain Locus of Control Scale OR MPRCQ OR MPRCQ2 OR pain stages of change questionnaire OR PSOCQ OR American Pain Society Pain Taxonomy OR AAPT OR "screener and opioid assessment for patients with pain" OR SOAPP OR MOPAT OR Overall benefit of analgesic score OR OBAS OR MMPAP OR Integrated Pain Quantification Index OR IPQI OR UAB Pain Behavior Scale OR "Pain and discomfort module" OR PDM OR Pain Experience Scale OR graded chronic pain scale OR GCPS OR chronic pain grade questionnaire OR CPGQ OR chronic pain grade scale OR CPGS OR Breakthrough Pain Assessment Tool OR BAT OR Richards Assessment of Pain OR RAP OR Mainz Pain Staging System OR MPSS OR Composite Pain Index OR CPI OR dallas pain questionnaire OR Clinically Aligned Pain Assessment OR CAPA OR Comprehensive Pain Evaluation Questionnaire OR CPEQ OR Survey of Pain Attitudes OR SOPA OR Multiaxial Assessment of Pain OR Multi-Facet Pain Survey OR MFPS OR "defense and veterans pain rating scale" OR DVPRS OR Pain Monitor OR Short Form-36 Bodily Pain Scale OR SF-36 Bodily Pain Scale OR SF-36 BPS OR Pain Impact Questionnaire OR PIQ OR Victorian Institute of Sports Assessment Achilles Questionnaire OR VISA-A OR ROwan Foot Pain Assessment Questionnaire OR ROFPAQ OR J-MAP OR Sickle Cell Disease Pain Burden Interview-Youth OR SCPBI-Y OR MHPQ OR Ocular Pain Assessment Survey OR OPAS OR Vulvar Pain Assessment Questionnaire OR "Migraine Tension-Type Headache and Neck Pain" OR M-TTH-NP OR "neck pain and disability scale" OR NPDS OR core outcome measures index OR COMI OR neck bournemouth questionnaire OR NBQ OR BQ OR Neuropathy Pain Scale OR PainDETECT OR "leeds assessment of neuropathic symptoms and signs" OR LANSS OR douleur neuropathique 4 OR DN4 OR Post abdominal surgery pain assessment scale OR PASPAS OR Edmonton Classification System for Cancer Pain OR pain assessment in advanced dementia OR PAINAD OR abbey pain scale OR APS OR Checklist of Nonverbal Pain Indicators OR CNPI OR Geriatric Pain Measure OR GPM OR GMPI OR Rheumatoid Arthritis Pain Scale OR RAPS OR "Measure of Intermittent and Constant Osteoarthritis Pain" OR ICOAP OR MPOPS OR critical care pain observation tool OR CPOT OR Angle Labor Pain Questionnaire OR A-LPQ).ab,ti.)

AND

(visual analog scale/ OR (unidimensional OR uni-dimensional OR monodimensional OR mono-dimensional OR faces pain scale OR FPS OR FACES OR Wong-Baker OR visual analog scal* OR visual analogue scal* OR VAS OR numeric rating scale OR numeric pain rating scale OR numerical pain rating scale OR numerical rating scale OR NRS OR NPRS OR vNRS OR analog pain scale OR analogue pain scale OR verbal rating scale OR VRS OR verbal pain score OR VPS OR verbal descriptor scale OR VDS OR verbal response scale).ab,ti.)

NOT ((exp adolescent/ OR exp child/ OR exp infant/) NOT exp adult/) NOT (exp animals/ NOT humans/)

Search narratives

- Hardly any of the scales that are indexed in Emtree have MeSH-terms.

CINAHL

(20211122; 129 hits)

((MH "Emergency medicine") OR (MH "emergency medical services") OR (MH "emergency services") OR (MH "trauma centers") OR (MH "emergency nursing" +) OR (MH "emergency treatment") OR (MH "emergency care" +) OR ((((TI emergency OR AB emergency) OR (TI emergencies OR AB emergencies) OR (TI trauma OR AB trauma) OR (TI accident OR AB accident) OR (TI urgency OR AB urgency) OR (TI urgencies OR AB urgencies)) N3 ((TI medicine OR AB medicine) OR (TI service* OR AB service*) OR (TI ward* OR AB ward*) OR (TI unit* OR AB unit*) OR (TI room* OR AB room*) OR (TI department* OR AB department*) OR (TI center* OR AB center*) OR (TI centre* OR AB centre*) OR (TI dispensary OR AB dispensary) OR (TI nursing OR AB nursing) OR (TI treatment* OR AB treatment*) OR (TI therapy OR AB therapy) OR (TI therapies OR AB therapies) OR (TI care OR AB care))) OR (TI emergicenter* OR AB emergicenter*) OR (TI emergicentre* OR AB emergicentre*) OR ((TI acute OR AB acute) N2 (TI care OR AB care))))

AND

((MH "Behavior Rating Scales") OR (MH "Brief Pain Inventory") OR (MH "McGill Pain Questionnaire") OR (MH "Memorial Pain Assessment Card") OR (TX multidimensional OR TX multi-dimensional OR TX "behavioral pain scale" OR TX "behavioural pain scale" OR TX "behavioral pain rating scale" OR TX "behavioural pain rating scale" OR TX "behavior pain scale" OR TX "behaviour pain scale" OR TX "behavior pain rating scale" OR TX "behaviour pain rating scale" OR TX BPS OR TX BPRS OR TX "brief pain inventory" OR TX BPI OR TX "carpal tunnel questionnaire" OR TX "carpal tunnel syndrome questionnaire" OR TX CTQ OR TX "CTS questionnaire" OR (TX constant N2 (TX murley OR TX scale OR TX score)) OR TX CMS OR TX "fear-avoidance beliefs questionnaire" OR TX "fear-avoidance belief questionnaire" OR TX "fear-avoidance questionnaire" OR TX "FAB questionnaire" OR TX FABQ OR TX "mcgill pain" OR TX "mcgill score" OR TX "mcgill test" OR TX MPQ OR TX SFMPQ OR TX "memorial pain assessment" OR TX "memorial pain card" OR TX "Migraine Disability Assessment" OR TX midas OR TX "neck disability index" OR TX "neck pain disability index" OR TX "patient rated wrist evaluation" OR TX "PRWE score" OR TX "shoulder pain and disability index" OR TX SPADI OR TX WHYMPI OR TX MPI OR TX MSS OR TX PEG OR TX MCPAC OR TX MAPS OR TX "Pain Locus of Control Scale" OR TX MPRCQ OR TX MPRCQ2 OR TX "pain stages of change questionnaire" OR TX PSOCQ OR TX "American Pain Society Pain Taxonomy" OR TX AAPT OR TX "screener and opioid assessment for patients with pain" OR TX SOAPP OR TX MOPAT OR TX "Overall benefit of analgesic score" OR TX OBAS OR TX MMPAP OR TX "Integrated Pain Quantification Index" OR TX IPQI OR TX "UAB Pain Behavior Scale" OR TX "Pain and discomfort module" OR TX PDM OR TX "Pain Experience Scale" OR TX "graded chronic pain scale" OR TX GCPS OR TX "chronic pain grade questionnaire" OR TX CPGQ OR TX "chronic pain grade scale" OR TX CPGS OR TX "Breakthrough Pain Assessment Tool" OR TX BAT OR TX "Richards Assessment of Pain" OR TX RAP OR TX "Mainz Pain Staging System" OR TX MPSS OR TX "Composite Pain Index" OR TX CPI OR TX "dallas pain questionnaire" OR TX "Clinically Aligned Pain Assessment" OR TX CAPA OR TX "Comprehensive Pain Evaluation Questionnaire" OR TX CPEQ OR TX "Survey of Pain Attitudes" OR TX SOPA OR TX "Multiaxial Assessment of Pain" OR TX "Multi-Facet Pain Survey" OR TX MFPS OR TX "defense and veterans pain rating scale" OR TX DVPRS OR TX "Pain Monitor" OR TX "Short Form-36 Bodily Pain Scale" OR TX "SF-36 Bodily Pain Scale" OR TX "SF-36 BPS" OR TX "Pain Impact Questionnaire" OR TX PIQ OR TX "Victorian Institute of Sports Assessment Achilles Questionnaire" OR TX VISA-A OR TX "ROwan Foot Pain Assessment Questionnaire" OR TX ROFPAQ OR TX J-MAP OR TX "Sickle Cell Disease Pain Burden Interview-Youth" OR TX SCPBI-Y OR TX MHPQ OR TX "Ocular Pain Assessment Survey" OR TX OPAS OR TX "Vulvar Pain Assessment Questionnaire" OR TX "Migraine Tension-Type Headache and Neck Pain" OR TX M-TTH-NP OR TX "neck pain and disability scale" OR TX NPDS OR TX "core outcome measures index" OR TX COMI OR

TX "neck bournemouth questionnaire" OR TX NBQ OR TX BQ OR TX "Neuropathy Pain Scale" OR TX PainDETECT OR TX "leeds assessment of neuropathic symptoms and signs" OR TX LANSS OR TX "douleur neuropathique 4" OR TX DN4 OR TX "Post abdominal surgery pain assessment scale" OR TX PASPAS OR TX "Edmonton Classification System for Cancer Pain" OR TX "pain assessment in advanced dementia" OR TX PAINAD OR TX "abbey pain scale" OR TX APS OR TX "Checklist of Nonverbal Pain Indicators" OR TX CNPI OR TX "Geriatric Pain Measure" OR TX GPM OR TX GMPI OR TX "Rheumatoid Arthritis Pain Scale" OR TX RAPS OR TX "Measure of Intermittent and Constant Osteoarthritis Pain" OR TX ICOAP OR TX MPOPS OR TX "critical care pain observation tool" OR TX CPOT OR TX "Angle Labor Pain Questionnaire" OR TX A-LPQ))

AND

((MH "Wong-Baker FACES Pain Rating Scale") OR (MH "visual analog scaling") OR (TX unidimensional OR TX uni-dimensional OR TX monodimensional OR TX mono-dimensional OR TX "faces pain scale" OR TX FPS OR TX FACES OR TX Wong-Baker OR TX "visual analog scal*" OR TX "visual analogue scal*" OR TX VAS OR TX "numeric rating scale" OR TX "numeric pain rating scale" OR TX "numerical pain rating scale" OR TX "numerical rating scale" OR TX NRS OR TX NPRS OR TX vNRS OR TX "analog pain scale" OR TX "analogue pain scale" OR TX "verbal rating scale" OR TX VRS OR TX "verbal pain score" OR TX VPS OR TX "verbal descriptor scale" OR TX VDS OR TX "verbal response scale"))

NOT ((MH "Child" OR MH "Adolescence") NOT MH "Adult")

Search narrative

- The acronym ED cannot be searched on EBSCOhost (produces an error)
- The multidimensional/unidimensional scale concepts were searched in the TX (all text) field rather than title + abstract to also search them in the full texts.

PMC

("Emergency medicine"[Mesh:noexp] OR "emergency medical services"[Mesh:noexp] OR "emergency service, hospital"[Mesh] OR "emergency nursing"[Mesh:noexp] OR "emergency treatment"[Mesh:noexp] OR (((emergency[ti] OR emergencies[ti] OR trauma[ti] OR accident[ti] OR urgency[ti] OR urgencies[ti] OR emergency[ab] OR emergencies[ab] OR trauma[ab] OR accident[ab] OR urgency[ab] OR urgencies[ab]) AND (medicine[ti] OR service*[ti] OR ward*[ti] OR unit*[ti] OR room*[ti] OR department*[ti] OR center*[ti] OR centre*[ti] OR dispensary[ti] OR nursing[ti] OR treatment*[ti] OR therapy[ti] OR therapies[ti] OR care[ti] OR medicine[ab] OR service*[ab] OR ward*[ab] OR unit*[ab] OR room*[ab] OR department*[ab] OR center*[ab] OR centre*[ab] OR dispensary[ab] OR nursing[ab] OR treatment*[ab] OR therapy[ab] OR therapies[ab] OR care[ab])) OR emergicenter*[ti] OR emergicentre*[ti] OR acute care[ti] OR emergicenter*[ab] OR emergicentre*[ab] OR acute care[ab]))

AND

((multidimensional[tw] OR multi-dimensional[tw] OR "behavioral pain scale"[tw] OR "behavioural pain scale"[tw] OR "behavioral pain rating scale"[tw] OR "behavioural pain rating scale"[tw] OR "behavior pain scale"[tw] OR "behaviour pain scale"[tw] OR "behavior pain rating scale"[tw] OR "behaviour pain rating scale"[tw] OR BPRS[tw] OR "brief pain inventory"[tw] OR BPI[tw] OR "carpal tunnel questionnaire"[tw] OR "carpal tunnel syndrome questionnaire"[tw] OR "CTS

questionnaire"[tw] OR constant murley[tw] OR contant scale[tw] OR constant score[tw] OR "fear-avoidance beliefs questionnaire"[tw] OR "fear-avoidance belief questionnaire"[tw] OR "fear-avoidance questionnaire"[tw] OR "FAB questionnaire"[tw] OR FABQ[tw] OR "mcgill pain"[tw] OR "mcgill score"[tw] OR "mcgill test"[tw] OR MPQ[tw] OR SFMPQ[tw] OR "memorial pain assessment"[tw] OR "memorial pain card"[tw] OR "Migraine Disability Assessment"[tw] OR midas[tw] OR "neck disability index"[tw] OR "neck pain disability index"[tw] OR "patient rated wrist evaluation"[tw] OR "PRWE score"[tw] OR "shoulder pain and disability index"[tw] OR SPADI[tw] OR WHYMPI[tw] OR MPI[tw] OR MSS[tw] OR MCPAC[tw] OR "Pain Locus of Control Scale"[tw] OR MPRCQ[tw] OR MPRCQ2[tw] OR "pain stages of change questionnaire"[tw] OR PSOCQ[tw] OR "American Pain Society Pain Taxonomy"[tw] OR AAPT[tw] OR "screener and opioid assessment for patients with pain"[tw] OR SOAPP[tw] OR MOPAT[tw] OR "Overall benefit of analgesic score"[tw] OR OBAS[tw] OR MMPAP[tw] OR "Integrated Pain Quantification Index"[tw] OR IPQI[tw] OR "UAB Pain Behavior Scale"[tw] OR "Pain and discomfort module"[tw] OR PDM[tw] OR "Pain Experience Scale"[tw] OR "graded chronic pain scale"[tw] OR GCPS[tw] OR "chronic pain grade questionnaire"[tw] OR CPGQ[tw] OR "chronic pain grade scale"[tw] OR CPGS[tw] OR "Breakthrough Pain Assessment Tool"[tw] OR "Richards Assessment of Pain"[tw] OR "Mainz Pain Staging System"[tw] OR MPSS[tw] OR "Composite Pain Index"[tw] OR "dallas pain questionnaire"[tw] OR "Clinically Aligned Pain Assessment"[tw] OR CAPA[tw] OR "Comprehensive Pain Evaluation Questionnaire"[tw] OR CPEQ[tw] OR "Survey of Pain Attitudes"[tw] OR SOPA[tw] OR "Multiaxial Assessment of Pain"[tw] OR "Multi-Facet Pain Survey"[tw] OR MFPS[tw] OR "defense and veterans pain rating scale"[tw] OR DVPRS[tw] OR "Pain Monitor"[tw] OR "Short Form-36 Bodily Pain Scale"[tw] OR "SF-36 Bodily Pain Scale"[tw] OR "SF-36 BPS"[tw] OR "Pain Impact Questionnaire"[tw] OR PIQ[tw] OR "Victorian Institute of Sports Assessment Achilles Questionnaire"[tw] OR VISA-A[tw] OR "ROwan Foot Pain Assessment Questionnaire"[tw] OR ROFPAQ[tw] OR J-MAP[tw] OR "Sickle Cell Disease Pain Burden Interview-Youth"[tw] OR SCPBI-Y[tw] OR MHPQ[tw] OR "Ocular Pain Assessment Survey"[tw] OR OPAS[tw] OR "Vulvar Pain Assessment Questionnaire"[tw] OR "Migraine Tension-Type Headache and Neck Pain"[tw] OR M-TTH-NP[tw] OR "neck pain and disability scale"[tw] OR NPDS[tw] OR "core outcome measures index"[tw] OR COMI[tw] OR "neck bournemouth questionnaire"[tw] OR NBQ[tw] OR BQ[tw] OR "Neuropathy Pain Scale"[tw] OR PainDETECT[tw] OR "leeds assessment of neuropathic symptoms and signs"[tw] OR LANSS[tw] OR "douleur neuropathique 4"[tw] OR DN4[tw] OR "Post abdominal surgery pain assessment scale"[tw] OR PASPAS[tw] OR "Edmonton Classification System for Cancer Pain"[tw] OR "pain assessment in advanced dementia"[tw] OR PAINAD[tw] OR "abbey pain scale"[tw] OR "Checklist of Nonverbal Pain Indicators"[tw] OR CNPI[tw] OR "Geriatric Pain Measure"[tw] OR GPM[tw] OR GMPI[tw] OR "Rheumatoid Arthritis Pain Scale"[tw] OR RAPS[tw] OR "Measure of Intermittent and Constant Osteoarthritis Pain"[tw] OR ICOAP[tw] OR MPOPS[tw] OR "critical care pain observation tool"[tw] OR CPOT[tw] OR "Angle Labor Pain Questionnaire"[tw] OR A-LPQ[tw]))

AND

("visual analog scale"[Mesh] OR (unidimensional[tw] OR uni-dimensional[tw] OR monodimensional[tw] OR mono-dimensional[tw] OR "faces pain scale"[tw] OR FPS[tw] OR Wong-Baker[tw] OR "visual analog scal*"[tw] OR "visual analogue scal*"[tw] OR "numeric rating scale"[tw] OR "numeric pain rating scale"[tw] OR "numerical pain rating scale"[tw] OR "numerical rating scale"[tw] OR NPRS[tw] OR vNRS[tw] OR "analog pain scale"[tw] OR "analogue pain scale"[tw] OR "verbal rating scale"[tw] OR VRS[tw] OR "verbal pain score"[tw] OR VPS[tw] OR "verbal descriptor scale"[tw] OR VDS[tw] OR "verbal response scale"[tw]))

NOT ((adolescent[Mesh] OR child[Mesh] OR infant[Mesh]) NOT adult[Mesh]) NOT (animals[Mesh] NOT humans[Mesh:noexp])

Search narrative

- The Emergency Department concept was searched in tiab fields ([ti] OR [ab] in PMC)
- The multidimensional/unidimensional scale concepts were searched in the [tw] (all text) field rather than title + abstract to also search them in the full texts.
- Because of a high noise, those scale acronyms that produced an increase of greater than 10 hits were removed from the search string
